# Therapeutic Targeting of Immune Cell Autophagy in Multiple Sclerosis: Russian Roulette or Silver Bullet?

**DOI:** 10.3389/fimmu.2021.724108

**Published:** 2021-08-31

**Authors:** Guan Yang, Luc Van Kaer

**Affiliations:** Department of Pathology, Microbiology and Immunology, Vanderbilt University School of Medicine, Nashville, TN, United States

**Keywords:** multiple sclerosis, experimental autoimmune encephalomyelitis (EAE), autophagy, LC3-associated phagocytosis (LAP), immune cells, therapy

## Abstract

Multiple sclerosis (MS) is a chronic inflammatory disease of the central nervous system (CNS) in which the immune system damages the protective insulation surrounding nerve fibers that project from neurons. The pathological hallmark of MS is multiple areas of myelin loss accompanied by inflammation within the CNS, resulting in loss of cognitive function that ultimately leads to paralysis. Recent studies in MS have focused on autophagy, a cellular self-eating process, as a potential target for MS treatment. Here, we review the contribution of immune cell autophagy to the pathogenesis of experimental autoimmune encephalomyelitis (EAE), the prototypic animal model of MS. A better understanding of the role of autophagy in different immune cells to EAE might inform the development of novel therapeutic approaches in MS and other autoimmune and inflammatory diseases.

## Introduction

Multiple sclerosis (MS) is a demyelinating disease of the central nervous system (CNS) with a critical role of autoreactive CD4^+^ T cells and myeloid cells in its disease pathogenesis (1, 2). Although the precise causes of MS remain unclear, genetic as well as environmental factors such as low vitamin D levels, smoking, and obesity are critical contributors (2). A characteristic feature of MS is demyelination of neurons in the CNS, resulting in loss of cognitive function and ultimately leading to paralysis. Globally, approximately 2-3 million people suffer from MS (2).

The “holy grail” for treatment of MS and other autoimmune diseases is to restore normal immune tolerance against the inciting self-antigens without disrupting natural and induced immunity against infectious agents and tumors (3). However, effective disease-specific treatments that selectively dampen autoantigen-specific immune responses are currently unavailable, whereas broadly disease-modifying treatments leave patients vulnerable to infections and possibly cancer (4). Thus, there is an urgent need to explore new avenues for treating MS and other autoimmune diseases. One approach with considerable promise is to target the cellular self-degradation process called macroautophagy that controls immune homeostasis and immune-mediated disease (5, 6).

Autophagy is a highly conserved cellular catabolism pathway that has been defined as an ‘auto-digestive’ process that promotes the degradation of long-lived or damaged cytoplasmic proteins and organelles by lysosomes (7, 8). The resulting products are then recycled for use as substrates in metabolic and biosynthetic pathways. Three different types of autophagy have been described based on how the particular cargo is delivered to lysosomes: macroautophagy, microautophagy, and chaperone-mediated autophagy (7). Macroautophagy delivers cytoplasmic cargo to lysosomes for degradation; microautophagy directly engulfs cytosolic components for lysosomal degradation; and chaperone-mediated autophagy translocates targeted proteins to lysosomes *via* binding to a complex that includes the chaperone HSPA8/HSC70 (heat shock cognate 71 kDa protein). As there is limited information about the possible contribution of microautophagy or chaperone-mediated autophagy in CNS autoimmunity, this review focuses on macroautophagy, hereafter referred to as autophagy.

**Figure 1:**
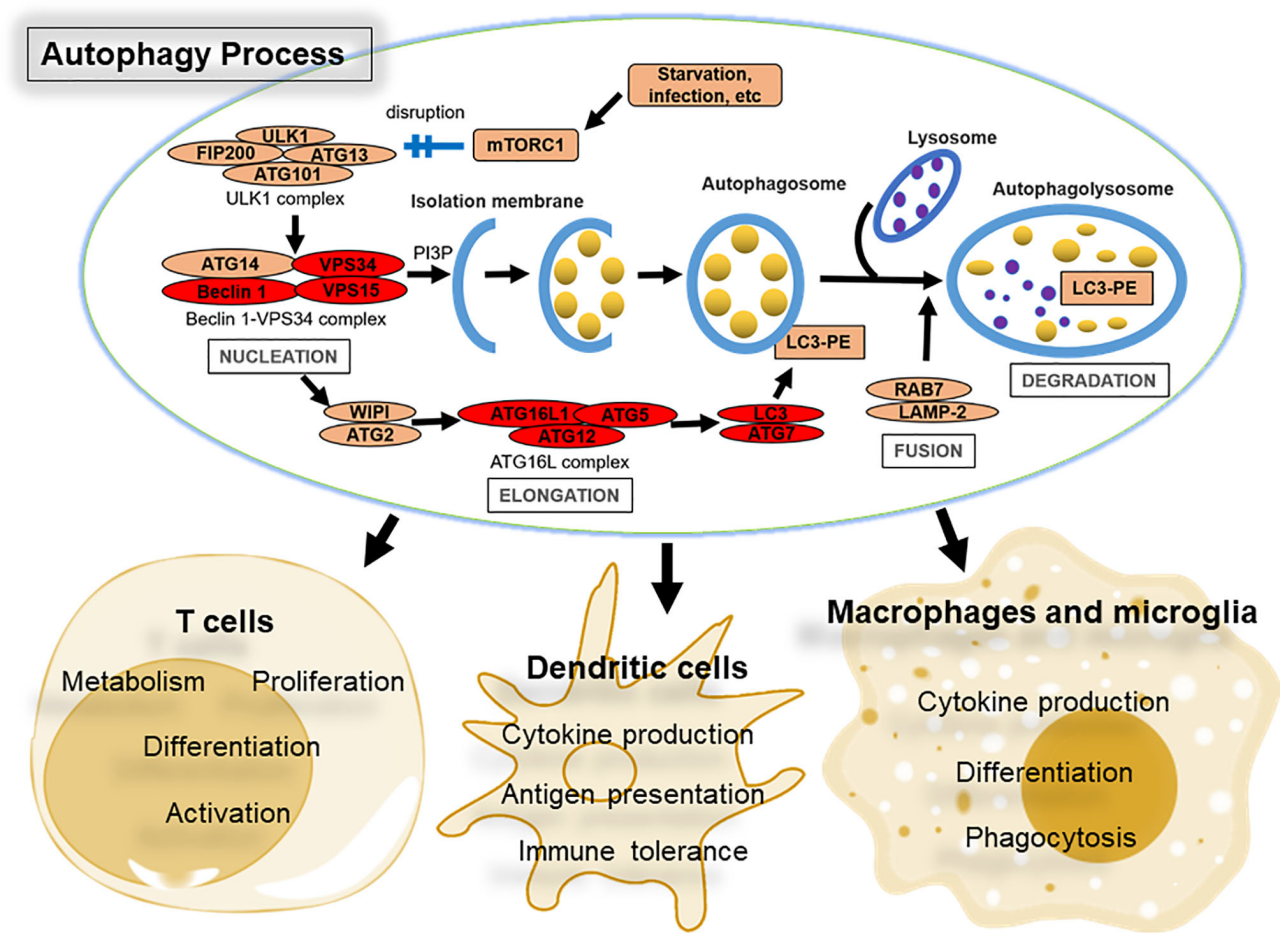
Overview of the autophagy pathway and its role in immune cells that control CNS autoimmunity. The four major steps in the autophagy process (nucleation, elongation, fusion, and degradation), together with key protein complexes and core factors, are indicated. Briefly, autophagy is initiated by nutrient starvation and other stress signals leading to the dissociation of the mechanistic target of rapamycin (mTOR) from the mTOR substrate complex (ULK1/2, ATG13, RB1CC1/FIP200, and ATG101). This dissociation leads to the recruitment of the class III phosphatidylinositol 3-kinase (PtdIns3K) complex, which includes at least BECN1/Beclin 1, PIK3C3/VPS34, PIK3R4/VPS15, and ATG14L that phosphorylates phosphatidylinositol to generate phosphatidylinositol-3-phosphate (PtdIns3P or PI3P). The ATG12-ATG5-ATG16L1 complex mediates the formation of pre-autophagosome structures. During this process, LC3 is conjugated to phosphatidylethanolamine with the assistance of ATG4, ATG7, and ATG3 (E2-like ubiquitin-conjugating enzymes) to generate LC3-II (LC3-PE), and associates with newly formed autophagosome membranes until they fuse with lysosomes to generate autophagolysosomes. The contents of autophagolysosomes are then degraded by lysosomal esterases, lipases, and proteases and recycled to build new cellular components and energy. Proteins shared by autophagy and LC3-associated phagocytosis (LAP) are highlighted in red. The main properties of immune cells impacted by autophagy during MS or EAE are listed.

Autophagy involves a variety of protein complexes composed of autophagy-related (ATG) gene products that were originally identified in yeast but are largely conserved in mammals (9, 10) (**Figure 1**). The morphological hallmark of autophagy is formation of the autophagosome, which is a double-membrane vesicle generated in a step-wise manner: nucleation, elongation, fusion, and degradation (7). Autophagy is initiated by nutrient starvation leading to the dissociation of the mechanistic target of rapamycin (mTOR) from the mTOR substrate complex (composed of ULK1/2, ATG13, RB1CC1/FIP200, and ATG101). This dissociation leads to the recruitment of the class III phosphatidylinositol 3-kinase (PtdIns3K) complex, which contains BECN1/Beclin 1, PIK3C3/VPS34, PIK3R4/VPS15, ATG14, NRBF2, UVRAG and possibly other factors, and phosphorylates phosphatidylinositol to generate phosphatidylinositol-3-phosphate (PtdIns3P), a phospholipid critical for membrane trafficking. The generation of PtdIns3P triggers autophagosomal maturation through recruitment of two ubiquitin-like proteins, which belong to the ATG12 and ATG8 family of ubiquitin-like proteins (including the microtubule-associated protein 1A/1B-light chain 3 [LC3] and GABARAP subfamilies). The ATG12-ATG5-ATG16L1 complex, in conjunction with ATG9, mediates the formation of pre-autophagosome structures. During this process, LC3 is conjugated to phosphatidylethanolamine with the assistance of ATG4, ATG7, and ATG3 (these are all E2-like ubiquitin-conjugating enzymes) to generate LC3-II, and associates with newly formed autophagosome membranes until they fuse with lysosomes. The generation of LC3-II is frequently used for monitoring autophagy (11, 12). Upon fusion with lysosomal membranes forming autophagolysosomes, the engulfed cytoplasmic cargo is degraded by lysosomal esterases, lipases, and proteases and recycled to build new cellular components and energy (13).

Autophagy plays a prominent role in directly regulating immune cell responsiveness to a variety of stimuli (14), and defects in this process have been linked to multiple human diseases, including inflammatory bowel disease, metabolic syndrome, cardiovascular diseases, infectious disorders, cancer, and neurodegeneration (9, 10). In this review, we summarize the aberrant changes in autophagy that occur in MS and its animal model, experimental autoimmune encephalomyelitis (EAE) (15, 16) (these alterations are summarized in **Table 1**), and how autophagy might be manipulated to influence MS disease pathogenesis.

**Table 1:** Autophagy is altered in MS and EAE.

CNS autoimmunity	Sample source	Autophagy-related protein	Reference
Relapsing-remitting MS	Peripheral T cells	*ATG5* ↑	(17)
Secondary progressive MS	Postmortem brain tissue	*ATG5* ↑	(17)
MS	Serum and cerebrospinal fluid	ATG5 and Parkin ↑	(18)
MS	Serum	ATG16L2 ↓	(19)
MS	Blood	*ATG16L2*, *ATG9A*, *FAS*, *GAA*, *HGS*, and *RAB24* ↓	(20)
		*ULK1*, *PIK3R1*, *BLC2*, *FOXO1*, *HTT*, and *RGS19* ↑	
EAE	Blood	*Atg5* ↑ ATG5 ↑	(17)
EAE	Spinal cord	p62 ↓; Early stage LC3-II ↑, peak stage LC3-II →	(21)
EAE	Spleen	p62 ↓	(22)
EAE	Monocyte-derived myeloid cells in the CNS	*Ulk1*, *Ulk2*, *Rb1cc1, Pik3c3* and *Becn1*, *Atg3*, *Atg5*, *Atg7*, *Gabarap* and *Map1lc3bp* ↑	(22)

↑, increased; ↓, decreased; →, unaltered.

## Autophagy Is Altered in MS

MS is associated with dysregulation of autophagic processes. Transcriptional analysis of peripheral T cells from patients with active relapsing-remitting MS (RRMS) and postmortem brain tissue from patients with secondary progressive MS revealed significantly elevated *ATG5* expression compared to non-disease controls (17). Fluorescent microscopy revealed strong ATG5 protein expression by T cells in the brain of RRMS patients, suggesting that encephalitogenic T cells are a significant source of ATG5 expression (17). A recent study also found enhanced serum and cerebrospinal fluid ATG5 and Parkin (a cytosolic E3 ubiquitin ligase mutated in Parkinson’s disease that promotes mitochondrial autophagy) levels in MS patients compared to patients with other inflammatory or non-inflammatory neurological diseases (18). Autophagy induction in circulating cells, especially T cells, may protract their survival, contributing to the pathogenesis of MS. Expression of ATG16L2, an isoform of ATG16L that inhibits ATG12-ATG5-ATG16L1 complex formation, was reduced in the serum of MS patients and this reduction was suggested to be mechanistically linked to defective T cell homeostasis in these patients (19). Consistent with this finding, another study that re-analyzed publicly available transcriptome data showed that *ATG16L2* gene expression is downregulated whereas *ULK1* gene expression is upregulated in blood samples of MS patients (20). Indeed, transcripts for 29 out of 78 ATGs analyzed in this study were significantly altered in MS patients, with 14 genes downregulated (including *ATG16L2*, *ATG9A*, *FAS*, *GAA*, *HGS*, and *RAB24*) and 15 genes upregulated (including *ULK1*, *PIK3R1*, *BLC2*, *FOXO1*, *HTT*, and *RGS19*) (20), suggesting the involvement of ATG gene products in MS pathogenesis.

## Autophagy Is Altered in EAE

The most commonly used animal model of MS is EAE, which can be actively induced through subcutaneous immunization of myelin-derived antigens (white matter homogenate, purified proteins, or peptides) suspended in complete Freund’s adjuvant, followed by injection of pertussis toxin to break the blood-brain barrier (15, 16). The pathogenesis of actively-induced EAE in mice consists of an induction and an effector phase. EAE can also be induced by adoptive transfer of activated myelin-specific CD4^+^ T cells into unimmunized mice, which models the effector phase of EAE (23).

In mice immunized with the myelin oligodendrocyte glycoprotein (MOG) peptide (35-55) (MOG_35-55_), *Atg5* transcript and ATG5 protein levels in peripheral blood positively correlated with EAE disease severity (17). Another study found reduced levels of the autophagy substrate sequestosome (SQSTM1, also known as p62) in the spinal cord at all stages of EAE (21), whereas LC3-II levels were increased at the early disease stage and returned to normal levels at the peak of disease (21). Similarly, our previous study revealed decreased p62 expression in spleen tissue and ongoing autophagy flux in splenic CD11b^+^ myeloid cells and CD4^+^ T cells of mice with EAE (22). We also observed increased expression of genes involved in different stages of the autophagic process (initiation: *Ulk1*, *Ulk2*, and *Rb1cc1;* nucleation: *Pik3c3* and *Becn1*; elongation: *Atg3*, *Atg5*, and *Atg7*; and fusion: *Gabarap* and *Map1lc3bp*) in monocyte-derived myeloid cells obtained from the CNS of mice with EAE (22).

These results are all consistent with induction of autophagy during MS and EAE development. However, these alterations in autophagy are not specific to MS and EAE, as abnormal autophagy activity also has been implicated in many other autoimmune conditions (5).

## Autophagy Manipulation in Immune Cells Alters EAE Pathogenesis

Autophagy has a central role in the development and function of many immune cell subsets that contribute to the pathophysiology of MS and EAE (24). A recent study employing single-cell RNA sequencing and intravital microscopy revealed expanded immune cells (mostly lymphocytes and myeloid cells) in the CNS of mice with EAE (25). Here we review how manipulation of autophagy levels in the key immune cell types that control the pathogenesis of EAE alters disease progression.

### T Lymphocytes

T cells are the major cellular contributors to the pathogenesis of MS (26, 27). The key role of T cells, especially CD4^+^ T cells, in MS has been confirmed in the EAE model. Briefly, peripheral T cells are primed to CNS autoantigens and then cross the blood-brain barrier to activate microglia and macrophages. In concert, these myelin sheath-targeting T cells infiltrate the CNS to induce death of myelin-producing oligodendrocytes and directly damage the myelin sheath around nerve fibers, leading to the loss of axons and neurons. Autophagy is markedly induced in activated CD4^+^ T cells and plays a critical role in the induction of autoreactive T cells. However, the function of T cell autophagy in EAE is under debate.

Several research groups including our own found that inhibition of autophagy in T cells protects mice from EAE development. Mice with T cell-specific ablation of *Pik3c3* or *Becn1* failed to mount autoimmune responses and were completely resistant to active EAE induction (28, 29). Mechanistically, these animals exhibited severely impaired MOG-reactive T helper (Th)1 and Th17 responses. These defects in Th cell differentiation in autophagy-deficient T cells were due to inhibited degradation of the procaspase-8/p62 signaling protein complex in activated T cells leading to apoptosis (28). Similarly, mice with T cell-specific deletion of *Atg7* were also completely protected from active EAE induction (30). T cells from the latter animals were hyporesponsive to antigenic stimulation and failed to accumulate in the spinal cord following EAE induction (30). To exclude effects mediated by defective T cell development, we adoptively transferred tamoxifen-treated splenic T cells derived from MOG_35-55_-immunized *pik3c3^f/f^;Rosa26-CreER^T2^* mice (the *Pik3c3* gene in these animals can be temporally deleted by treatment with tamoxifen, which binds to the estrogen receptor [ER] T2 to release Cre recombinase from the cytoplasm to the nucleus) at 10 days after immunization to wild-type (WT) animals, resulting in profoundly impaired EAE (29). Together, these results provide evidence that T cell autophagy is required to establish efficient T cell responses and to break tolerance against CNS antigens during EAE.

Each of the T cell studies discussed thus far employed a *Cre* gene controlled by the *Cd4* gene promoter/enhancer that is expressed starting from the late double-negative (CD4^-^CD8^-^) to double-positive (CD4^+^CD8^+^) stage of T cell development in the thymus. Another report conditionally deleted *Atg7* in T cells using a *Cre* gene driven by the proximal *Lck* promoter that is expressed starting from the double-negative thymocyte stage, and these animals developed normal levels of EAE (31). The latter animals also exhibited relatively normal frequencies of CD3^+^ T cells in peripheral lymphoid organs (31). In contrast, several other groups reported significantly decreased mature CD3^+^ T cells both in percentage and absolute numbers in *atg7^f/f^;pLck-Cre* mice (32–34), a phenotype also found in *pik3c3^f/f^*;*Cd4*-*Cre*, *pik3c3^f/f^*;*pLck-Cre*, *becn1^f/f^*;*Cd4*-*Cre*, and *atg7^f/f^*;*Cd4*-*Cre* mice (28, 33, 35–37). The factors responsible for these apparent divergent T cell frequencies observed in different studies of conditional *Atg7* knockout animals using the same *pLck* promoter to drive *Cre* expression remain unclear and require further study.

### Macrophages and Microglia

The CNS contains a heterogeneous population of resident macrophages such as microglia, choroid plexus macrophages, meningeal macrophages, and perivascular macrophages (38). However, due to a lack of experimental approaches that could reveal this macrophage heterogeneity in EAE models, the roles of different subsets of CNS macrophages and their interactions with encephalitogenic T cells remain to be uncovered (39). It is generally acknowledged that macrophages and microglia are important mediators of CNS inflammation during EAE progression (40). In the early phase of EAE, macrophages and microglia exhibit an M1 (classically activated) phenotype, which causes the release of proinflammatory cytokines in association with promotion of T cell responses and myelin injury. During the resolving phase of EAE, M2 (alternatively activated) microglia and macrophages become dominant and facilitate tissue repair by releasing anti-inflammatory cytokines.

Autophagy is involved in many aspects of macrophage and microglia biology, ranging from cellular differentiation, polarization, phagocytosis, and cytokine production (41, 42). Therefore, autophagy could be targeted to manipulate the properties of macrophages and microglia in EAE.

Selective *Pik3c3*, *Atg5*, or *Atg7* deficiency in myeloid cells using a *Lyz2-Cre* transgene resulted in attenuated EAE disease severity (22, 43, 44). This protection was not due to alterations in the induction of pathogenic T cells, because mice with myeloid cell-specific *Pik3c3* or *Atg7* deficiency also exhibited reduced EAE after adoptive transfer of myelin-specific T cells from WT animals (22, 44). This attenuated EAE phenotype was associated with changes in many physical and cellular processes, including reduced breakdown of blood-spinal cord barrier permeability (43), decreased CNS immune cell infiltration (22, 43), reduced IL-1β production by myeloid cells (22), or stalling of Th17 cells in the lung *en route* to the CNS (44). Interestingly, no impairment of peripheral antigen-specific T cell activation was observed in myeloid cell-specific *Pik3c3-*, *Atg5-*, or *Atg7*-deficient mice (22, 43, 44). The importance of macrophage autophagy in EAE pathogenesis was further reinforced by the finding that adoptive transfer of WT macrophages into *pik3c3^f/f^;Lyz2-Cre* mice before EAE induction was able to overcome the resistance of these animals to EAE (22). In contrast to these findings, one group reported that deletion of *Atg7* in myeloid cells leads to a persistent EAE disease state that lacks the normal disease recovery phase (31). This lack of recovery was further found to be due to *Atg7* deficiency in microglia using tamoxifen-treated *atg7^fl/fl^*;*Cx3cr1^CreERT2^* mice, leading to defects in microglial tissue debris clearance in a manner dependent on noncanonical autophagy (31). Another recent study reported no impact on the development of EAE upon *Atg5* deletion in postnatal microglia using *Sall1-Cre* for conditional gene targeting (45). While reasons for these contrasting findings are unknown, these studies employed distinct *Cre* gene driver transgenic lines, suggesting differences in the subsets of myeloid lineage cells targeted in the different conditional knockout mice as a likely cause. Thus, it is paramount to elucidate the individual roles of distinct CNS macrophage subsets in EAE.

In addition to macrophages and some microglia, *Lyz2-Cre* can be expressed in multiple other cell lineages, including some neutrophils and dendritic cells (DCs) (46, 47). Thus, it will be of interest to investigate the role of autophagy in other myeloid lineage cells during EAE. Indeed, one of the aforementioned studies suggested that the protective effect against EAE in mice with a myeloid cell-specific deficiency of *Atg5* or *Atg7* may be contributed by defective neutrophil function (43), which correlated significantly with EAE development (48).

Together, these studies revealed an important role of autophagy in macrophages and microglia during EAE, although the conflicting reports describe ATG gene products as either anti- or pro-encephalitogenic. The direct and precise effect of autophagy-deficient myeloid cells on disease development warrants further investigation.

### Dendritic Cells

DCs are the most potent antigen-presenting cells (APCs) for priming of autoantigen-specific T cell responses during EAE. Autophagy influences antigen presentation by DCs and other APCs. For instance, DCs from patients expressing Crohn’s disease-associated *ATG16L1* or *NOD2* risk variants were defective in antigen presentation (49).

Treatment of murine DCs, macrophages and thymic DCs with 3-methyladenine (3-MA), a non-specific autophagy inhibitor, impaired the presentation of citrullinated peptides to T cells (50). In EAE models, ablation of *Atg7*, *Pik3c3*, or *Rb1cc1* in DCs significantly attenuated the onset of EAE upon active immunization by reducing *in vivo* priming of T cells (21, 51). In addition, *Atg16l1*-deficient DCs were less efficient in priming autoreactive T cells and, consequently, DC-specific *Atg16l1*-deficient mice failed to develop EAE upon receiving an adoptive transfer of primed T cells isolated from transgenic mice carrying a T cell receptor specific for the MOG_35–55_ peptide (2D2 T cells) (52). We further showed that *Pik3c3*- and *Rb1cc1*-deficient DCs are less efficient in presenting recombinant MOG_1-125_ protein (requires intracellular processing for its presentation) but not MOG_35-55_ peptide (does not require intracellular processing for its presentation) to 2D2 T cells (51). However, in another report where *Atg5* was disrupted in CD11c^+^ DCs, normal peripheral priming of CD4^+^ T cells following active immunization with myelin protein was found, which resulted in a modest (statistically nonsignificant) alleviation of EAE incidence rates and clinical scores (53). These investigators found that ATG5 is required for accumulation of myelin-specific CD4^+^ T cells within the CNS and that ablation of *Atg5* in DCs leads to complete protection from EAE in an adoptive transfer model (53). Collectively, these results suggest that the autophagic machinery in DCs is required for normal processing of extracellular MOG antigen to MHC class II-restricted T cells. However, the direct effect of autophagy-deficient DCs on EAE development has not been fully elucidated, partly due to limited understanding of the mechanisms that regulate myelin antigen processing *in vivo*. Notably, this type of exogenous processing of antigens for presentation to MHC class II-restricted T cells also has been described in other circumstances, such as during autophagy of damaged endosomes and during phagosome maturation following LC3 attachment (54–56). The incomplete disease protection afforded by conditionally knocking out these ATG genes in DCs could be due to incomplete *Cd11c-Cre*-mediated gene deletion in plasmacytoid DCs (57), which contribute significantly to T cell priming during EAE (58). Other types of APCs may also be involved in EAE development (59).

## Contribution of LC3-Associated Phagocytosis to EAE

**Figure 2:**
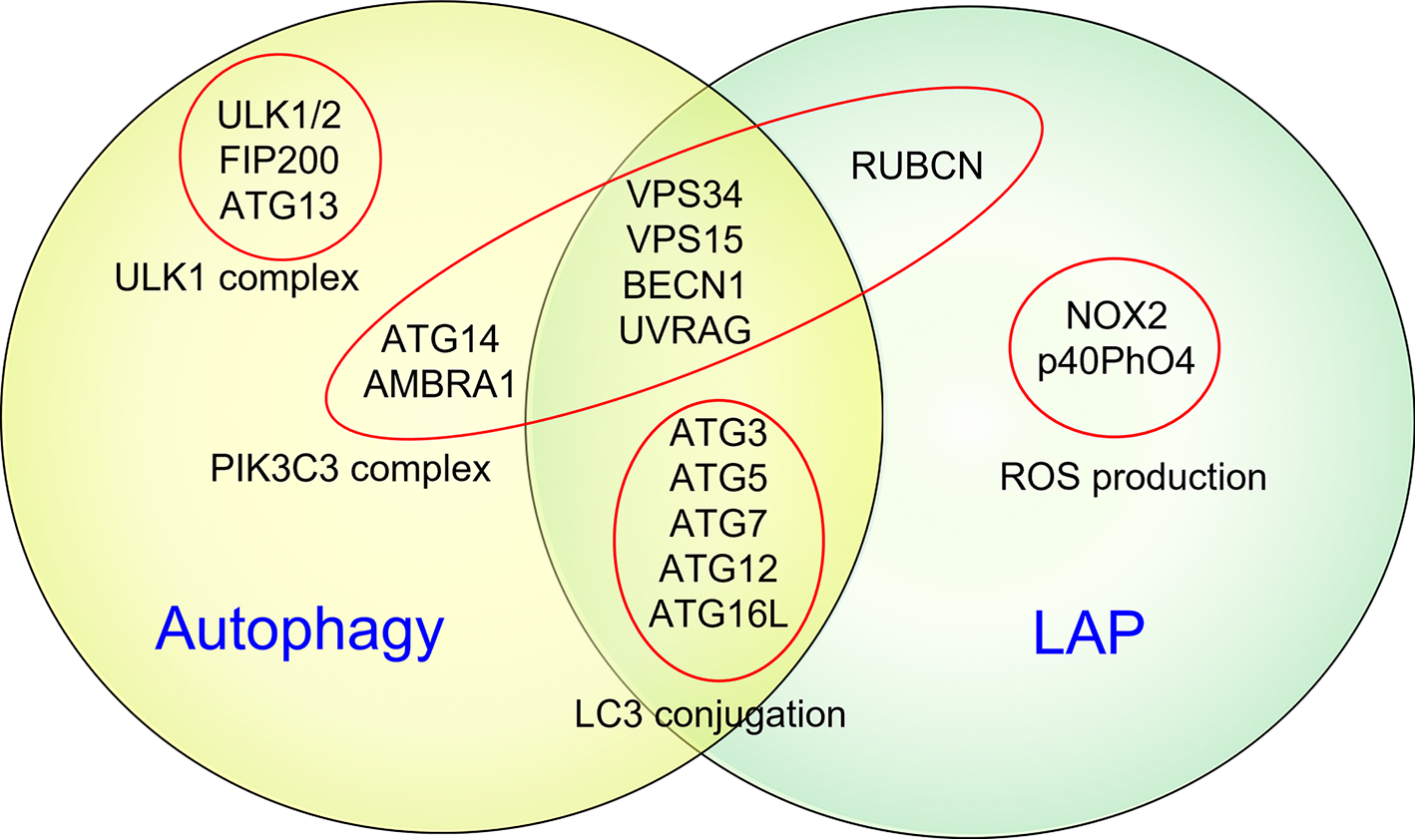
Autophagy and LAP share several key molecular regulators and machinery. It is now widely acknowledged that some common components of the autophagic machinery, especially constituents of the PIK3C3 complex, are shared by autophagy and LAP. Unlike autophagy, LAP is an autophagosome-independent process that occurs independently of the mTOR substrate complex (ULK1/2, ATG13, RB1CC1/FIP200, and ATG101) but relies on RUBCN, CYBB/NOX2, and the WD domain of ATG16L1, which are all dispensable for autophagy.

It is now widely acknowledged that most, if not all, components of the autophagy apparatus also mediate non-autophagic functions, including LC3-associated phagocytosis (LAP) (**Figure 2**), endocytosis, melanogenesis, cytokinesis, Golgi apparatus to endoplasmic reticulum transport, etc. (60). Of note, PIK3C3, BECN1, ATG7, ATG5, and ATG16L1 are all shared between autophagy and LAP (60). Unlike autophagy, LAP is an autophagosome-independent process that occurs independently of the mTOR substrate complex (ULK1/2, ATG13, RB1CC1/FIP200, and ATG101) but relies on RUBCN, CYBB/NOX2, and the tryptophan-aspartic acid (WD) domain of ATG16L1, which are all dispensable for autophagy (60). However, the role of LAP in pathological mechanisms of EAE remains controversial.

We examined the ability of LAP- and LC3-associated endocytosis (LANDO)-deficient but autophagy-sufficient (*rubcn* ^-/-^) mice to develop EAE and found disease similar to WT control mice, employing both active and passive EAE models (29, 51). Using mice with a DC-specific *Rubcn* deficiency, we further confirmed that DCs do not require LAP for stimulating autoreactive T cells and inducing EAE development (51). In contrast, Keller et al. found that DC-specific deletion of *Cybb* inhibits EAE clinical disease development in an adoptive transfer model, but does not affect actively induced EAE (61). However, it should be noted that CYBB may influence DC functions in various LAP-independent pathways (62, 63) and CYBB loss in DCs could be potentially compensated for by other NADPH oxidase family members, or possibly by other forms of reactive oxygen species as reported in other cell types (64, 65). Therefore, how myelin antigen is processed and presented by DCs in the CNS awaits careful investigation to further elucidate the relative role of autophagy, LAP, and other cellular processes during EAE.

## Modulation of EAE by Targeting Autophagy

Based on the immunomodulatory activities of autophagy and its potential role in the development of MS and EAE, several studies have assessed the capacity of autophagy modulators to influence EAE. While promising, divergent results have been obtained.

### Autophagy Activation

Rapamycin is a potent inhibitor of mTOR by forming a complex with the immunophilin FK506-binding protein 12 (FKBP12), which then stabilizes the association of mTOR with its regulatory associated protein raptor to inhibit the kinase activity of mTOR (66). Rapamycin induces autophagy both *in vivo* and *in vitro*. Treatment with rapamycin has significant benefits on EAE (67, 68). Although rapamycin is a strong activator of autophagy and has been tested as an immunosuppressant, caution should be taken in interpreting the activation of autophagy as immunosuppressive activity, as accumulating evidence suggests that activation of autophagy can promote the survival and pathogenicity of autoreactive T cells (69). By promoting intracellular recycling, autophagy can confer stress resistance and sustain cell survival under adverse conditions, which may promote EAE pathogenesis. Thus, the efficacy of rapamycin against EAE may depend on its inhibitory effects on protein translation and metabolism (70) rather than autophagy activation.

### Autophagy Inhibition

Different laboratories have reported divergent effects of autophagy inhibition on the incidence and progression of EAE, raising questions about the therapeutic effects of these drugs in EAE. Treatment with chloroquine, which blocks the fusion of autophagosomes with lysosomes, at different stages of EAE development delays disease onset or severity (21, 71). Similarly, we recently observed delayed EAE disease progression in mice treated with a selective PIK3C3 inhibitor, SAR405 (22). Pretreatment of mesenchymal stem cells with the PtdIns3K inhibitor 3-MA improves the therapeutic effects of these cells against EAE (72). Interestingly, treatment with 3-MA exacerbates EAE development (73) and attenuates the protective effects of α7 nicotinic acetylcholine receptor activation on EAE (74). These opposing effects of 3-MA on EAE may reflect its capacity to inhibit not only PIK3C3 (a class III PtdIns3K) but also class I PtdIns3K, which might play opposing roles in autophagy (75). Additionally, class I PtdIns3K isoforms play roles in leukocyte function and inflammation, and their inhibition might therefore affect EAE *via* immune modulation as well as autophagy. These data also provide a reminder to remain cautious when interpreting findings obtained with non-selective autophagy inhibitors.

## Conclusions and Future Perspectives

The studies reviewed here identify autophagy as a promising therapeutic target in MS and other autoimmune diseases. Although pharmacological autophagy modulators offer feasible solutions, the available drugs are often not specific to autophagy (76). It is also worth noting that systemic autophagy inhibition may cause the aggregation of misfolded proteins in neurons, leading to neurodegenerative diseases (77). Since autophagy is a ubiquitous process, and distinct immune cell subsets exhibit differing autophagic activity, it would be beneficial to target autophagy modulators to specific immune cell types, including cells with pathogenic or suppressive functions. As autophagy levels differ during different stages of clinical MS, autophagy modulators could be employed during different disease stages to test their capacity to modify the course of MS disease. Finally, autophagy modulators could be combined with additional MS disease-modifying therapeutics (78, 79).

## Author Contributions

GY: Conceptualization, Writing-Original draft preparation. LVK: Conceptualization, Writing-Reviewing and Editing. All authors contributed to the article and approved the submitted version.

## Funding

Work in the authors’ lab was supported by grants from the NIH (AI139046 to LVK) and the National Multiple Sclerosis Society (60006625 to LVK).

## Conflict of Interest

The authors declare that the research was conducted in the absence of any commercial or financial relationships that could be construed as a potential conflict of interest.

## Publisher’s Note

All claims expressed in this article are solely those of the authors and do not necessarily represent those of their affiliated organizations, or those of the publisher, the editors and the reviewers. Any product that may be evaluated in this article, or claim that may be made by its manufacturer, is not guaranteed or endorsed by the publisher.

## Abbreviations

APCs, antigen-presenting cells; ATG, autophagy-related; CNS, central nervous system; DC, dendritic cell; EAE, experimental autoimmune encephalomyelitis; LAP, LC3-associated phagocytosis; LC3, microtubule-associated protein 1A/1B-light chain 3; MS, multiple sclerosis; PtdIns3K, class III phosphatidylinositol 3-kinase; PtdIns3P, phosphatidylinositol-3-phosphate; RRMS, relapsing-remitting MS; Th, T helper; 3-MA, 3-methyladenine.
